# In the December 2013 issue

**DOI:** 10.1590/S1980-57642013DN74000001

**Published:** 2013

**Authors:** Ricardo Nitrini

In this issue of Dementia *&* Neuropsychologia, we are publishing one review, ten original papers, one case report and the first paper inaugurating the History Note section of the journal, which shall be edited by Prof. Eliasz Engelhardt.

**Brucki** reviewed the epidemiology of mild cognitive impairment (MCI) in Brazil concluding that Brazilian data remain scarce hampering comparisons with other countries. Her findings should prompt investigators to undertake and publish investigations on the epidemiology of MCI in Brazil.

**Batista et al**. investigated the test-retest reliability of tests of the Brazilian version of the CERAD battery. Better retest scores were observed with a 20- day interval, suggesting that retest should be applied after longer intervals or by using alternate forms of the tests.

**Foss et al.** evaluated the performance of middle-aged and elderly Brazilian on the Mattis Dementia Rating Scale (DRS) to propose norms for improving the diagnostic accuracy of this scale. Age and educational level were taken into account as these two factors have strong correlation with DRS scores.

**Silagi et al.** investigated the effects of social exposure on communication in the elderly. The authors found that sociodemographic features were associated with communication habits (partners and communication time) and that time devoted to communication with friends may indicate cognitive difficulties

**Lima-Silva et al.** carried out the translation and cross-cultural adaptation of the Frontotemporal Dementia Rating Scale (FTD-FRS) for use in Brazil. This scale was considered suitable to aid in staging and determining disease progression for Brazilian- Portuguese-speaking patients with frontotemporal dementia.

**Souza-do-Carmo et al.** investigated renal function in elderly with and without Alzheimer’s disease (AD). Their conclusions were that patients with AD have lower glomerular filtration and a greater incidence of microalbuminuria, not associated with higher vascular risk factors. These are preliminary findings and further studies should be conducted to investigate the renal function of elderly with AD.

**Castro-Costa et al.** investigated the association between nutritional status and cognitive impairment in community-dwelling elderly. The authors found that cognitive impairment was associated with fat mass measures in the group aged between 60 and 69 yet with muscle mass measures in the group aged over 70.

**Rezende et al.** compared the Cognitive Abilities Screening Instrument-Short Form (CASI-S) with Mini-Mental State Examination (MMSE), and the Functional Activities Questionnaire for the diagnosis of dementia in illiterate elderly. The authors found a high correlation between the CASI-S and MMSE and suggested further studies to evaluate the use of CASI-S for dementia diagnosis in populations with a heterogeneous educational background.

**Marroni et al.** evaluated the normal values of cerebral perfusion using single-photon emission tomography (SPECT) with ^99m^Tc-ethyl cysteinate dimmer. This method may allow further quantification of SPECT data, specifically the comparison of patients with normal controls.

**Hur & Caixeta** evaluated more complex language tasks in two cases of semantic dementia, finding that the interpretations of proverbs and metaphors as well as comprehension of ironic statements were disturbed. The possible causes of these symptoms are discussed.

**Fortes et al**. reported two cases of neurosarcoidosis presenting as rapidly progressive dementia. The diagnostic criteria and differential diagnosis of this condition are discussed.

In this inaugural paper of the History Note section, **Engelhardt** reviewed the pioneering studies on basal formations of the brain, particularly of Meynert’s basal nucleus, emphasizing the works of Meynert and also of Reil, Gratiolet and Koelliker.

**Champs et al.** reported a case of a patient with progressive paraparesis and cognitive changes referred with the possible diagnosis of multiple sclerosis, whose serum and cerebrospinal fluid were positive for HTLV-I infection. The authors discuss the differential diagnosis between these two conditions emphasizing the dissimilar patterns of cognitive impairment.

**Ricardo Nitrini** Editor-in-Chief

