# Supplementary material for: In the December 2013 issue
Source: Dement Neuropsychol. 2013 Oct-Dec;7(4):361–2. doi: 10.1590/S1980-57642013DN74000001 (PMC5619495; doi:10.1590/S1980-57642013DN74000001)
Supplement: Related texts

# Other: HISTORY NOTE: A NEW SECTION

**Authors:** Eliasz Engelhardt

Historical Note is a new permanent section now being inaugurated in Dementia *&* Neuropsychologia.

One might ask: why study history of neurology and related issues, when history is largely regarded as events of the past that are outdated and no longer valued in practical terms?

The answer is clear: our present is based on our past, and we should be fully aware of where we have been, and where we are, before we venture into the future. Traditional sayings such as “If I have seen further it is by standing on the shoulders of giants”, among others, clearly denote this view.

Studying history develops a critical view of present achievements. History allows us to understand the pioneering researchers, their lives and struggles, their reasoning, their successes and failures, the relentless pursuit of their aims in an unabated fashion, their achievements, how they paved the way in a poorly charted brain, their egos…

To view past achievements as meritless is folly, as oftentimes, ill-described structures can become, sometimes decades or centuries later, advance techniques and flourishing functional systems.

History allows us to explore the roots of today’s ideas and practices and thus to appreciate both the ephemeral nature of knowledge and the need for continual learning. A sense of humility and of constructive criticism for personal endeavors develops in those who look to past achievements.

Some authors that have inspired these considerations should be cited: such as Cordell, 1904; Guthrie, 1945; Thornton, 1987; Finger, 2000; Koehler et al., 2000; Maienschein,2000; Gooday et al., 2008; Roche et al., 2009; Gomes and Teive, 2012.

The instructions for submitting manuscripts are very flexible: the main text may have up to 1,500 words, with an abstract, including keywords, containing about 150 words, references may number up to 30, and, optionally, up to 2 figures and 2 tables or boxes may be included. General additional information can be obtained from the site of the journal.

One should keep in mind that writing history requires the reading of original texts (primary) which represents a firsthand re-reading and re-interpretation of the writings, as it is known that very often the filter of time and of secondary works may distort or even miss out parts of the original findings, resulting in errors which may frequently be accepted as truths, and perpetuated for long periods.

Call for papers: After these reflections and instructions, the editor hereby invites all professionals from our neurological and cognitive community to submit a paper on history. Clearly, in view of the characteristics of this journal, the focus should center mainly on authors and their works that have influenced thinking on cognition, behavior and related neuroscientific issues.

Now is the time to begin.

**Eliasz Engelhardt** Editor, History Note section
